# Assessment of Clinicopathological Characteristics and Development of an Individualized Prognostic Model for Patients With Hepatoid Adenocarcinoma of the Stomach

**DOI:** 10.1001/jamanetworkopen.2021.28217

**Published:** 2021-10-05

**Authors:** Jian-Xian Lin, Zu-Kai Wang, Qing-Qi Hong, Peng Zhang, Zi-Zhen Zhang, Liang He, Quan Wang, Liang Shang, Lin-Jun Wang, Ya-Feng Sun, Zhi-Xiong Li, Jun-Jie Liu, Fang-Hui Ding, En-De Lin, Yong-An Fu, Shuang-Ming Lin, Jian-Wei Xie, Ping Li, Chao-Hui Zheng, Chang-Ming Huang

**Affiliations:** 1 Department of Gastric Surgery, Fujian Medical University Union Hospital, Fuzhou, China; 2 Key Laboratory of Ministry of Education of Gastrointestinal Cancer, Fujian Medical University, Fuzhou, China; 3 Department of Gastrointestinal Oncology Surgery, Xiamen Cancer Center, The First Affiliated Hospital of Xiamen University, Xiamen, China; 4 Department of Gastrointestinal Surgery, Union Hospital, Tongji Medical College, Huazhong University of Science and Technology, Wuhan, China; 5 Department of Gastrointestinal Surgery, Ren Ji Hospital, Shanghai Jiao Tong University School of Medicine, Shanghai, China; 6 Department of Gastrointestinal Surgery, First Hospital of Jilin University, Changchun, China; 7 Department of Gastrointestinal Surgery, Shandong Provincial Hospital Affiliated to Shandong First Medical University, Jinan, China; 8 The First Affiliated Hospital of Nanjing Medical University, Nanjing, China; 9 Department of Gastrointestinal Surgery, The Second Affiliated Hospital of Fujian Medical University, Quanzhou, China; 10 Gastrointestinal Surgery Unit 1, Teaching Hospital of Putian First Hospital of Fujian Medical University, Putian, China; 11 Gastrointestinal Department of The Sixth Affiliated Hospital of Sun Yat-sen University, Guangzhou, China; 12 General Surgery Department, The First Hospital of Lanzhou University, Lanzhou, China; 13 Department of General Surgery, Zhongshan Hospital Affiliated with Xiamen University, Xiamen, China; 14 Department of Gastrointestinal Surgery, Affiliated Quanzhou First Hospital to Fujian Medical University, Quanzhou, China; 15 Department of Gastrointestinal Surgery, Longyan First Hospital Affiliated with Fujian Medical University, Longyan, China

## Abstract

**Importance:**

Few studies have examined the clinicopathological characteristics and prognoses of patients with hepatoid adenocarcinoma of the stomach (HAS).

**Objective:**

To explore the clinicopathological characteristics and prognoses of patients with HAS and develop a nomogram to predict overall survival (OS).

**Design, Setting, and Participants:**

This prognostic study involved a retrospective analysis of data from 315 patients who received a diagnosis of primary HAS between April 1, 2004, and December 31, 2019, at 14 centers in China.

**Main Outcomes and Measures:**

OS and prognostic factors. Patients were randomly assigned to a derivation cohort (n = 220) and a validation cohort (n = 95). A nomogram was developed based on independent prognostic factors identified through a multivariable Cox mixed-effects model.

**Results:**

Among 315 patients with HAS (mean [SD] age, 61.9 [10.2] years; 240 men [76.2%]), 137 patients had simple HAS (defined as the presence of histologically contained hepatoid differentiation areas only), and 178 patients had mixed HAS (defined as the presence of hepatoid differentiation areas plus common adenocarcinoma areas). Patients with simple HAS had a higher median preoperative α-fetoprotein level than those with mixed HAS (195.9 ng/mL vs 48.9 ng/mL, respectively; *P* < .001) and a higher rate of preoperative liver metastasis (23 of 137 patients [16.8%] vs 11 of 178 patients [6.2%]; *P* = .003). The 3-year OS rates of patients with simple vs mixed HAS were comparable (56.0% vs 60.0%; log-rank *P* = .98). A multivariable Cox analysis of the derivation cohort found that the presence of perineural invasion (hazard ratio [HR], 2.13; 95% CI, 1.27-3.55; *P* = .009), preoperative carcinoembryonic antigen levels of 5 ng/mL or greater (HR, 1.72; 95% CI, 1.08-2.74; *P* = .03), and pathological node category 3b (HR, 3.72; 95% CI, 1.34-10.32; *P* = .01) were independent risk factors for worse OS. Based on these factors, a nomogram to predict postoperative OS was developed. The concordance indices of the nomogram (derivation cohort: 0.72 [95% CI, 0.66-0.78]; validation cohort: 0.72 [95% CI, 0.63-0.81]; whole cohort: 0.71 [95% CI, 0.66-0.76]) were higher than those derived using the American Joint Committee on Cancer’s *AJCC Cancer Staging Manual* (8th edition) pathological tumor-node-metastasis (pTNM) staging system (derivation cohort: 0.63 [95% CI, 0.57-0.69]; validation cohort: 0.65 [95% CI, 0.56-0.75]; whole cohort: 0.64 [95% CI, 0.59-0.69]) and those derived using a clinical model that included pTNM stage and receipt of adjuvant chemotherapy (derivation cohort: 0.64 [95% CI, 0.58-0.69]; validation cohort: 0.65 [95% CI, 0.56-0.75]; whole cohort: 0.64 [95% CI, 0.59-0.69]). Based on the nomogram cutoff of 10 points, the whole cohort was divided into high-risk and low-risk groups. The 3-year OS rate of patients in the high-risk group was significantly lower than that of patients in the low-risk group (29.7% vs 75.9%, respectively; log-rank *P* < .001), and the 3-year prognosis of high-risk and low-risk groups could be further distinguished into pTNM stage I to II (33.3% vs 80.2%; exact log-rank *P* = .15), stage III (34.3% vs 71.3%; log-rank *P* < .001), and stage IV (15.5% vs 70.3%; log-rank *P* = .009).

**Conclusions and Relevance:**

This study found that perineural invasion, preoperative carcinoembryonic antigen levels of 5 ng/mL or greater, and pathological node category 3b were independent risk factors associated with worse OS. An individualized nomogram was developed to predict OS among patients with HAS. This nomogram had good prognostic value and may be useful as a supplement to the current American Joint Committee on Cancer TNM staging system.

## Key Points

**Question:**

What are the clinicopathological characteristics and prognoses of patients with hepatoid adenocarcinoma of the stomach (HAS), and what is an accurate and efficient method of predicting overall survival (OS) among these patients?

**Findings:**

In this prognostic study of 315 patients with HAS, 3 risk factors (perineural invasion, preoperative carcinoembryonic antigen levels ≥5 ng/mL, and pathological node category 3b) were independently associated with worse OS among patients with HAS. Based on these risk factors, a nomogram to predict postoperative OS among patients with HAS was developed.

**Meaning:**

This study found that, among patients with HAS, perineural invasion, preoperative carcinoembryonic antigen levels, and pathological node category 3b were independently associated with OS; an individualized nomogram that was developed to predict postoperative OS had good prognostic value and may be useful as a supplement to the current tumor-node-metastasis staging system.

## Teaser

This prognostic study examines the clinicopathological characteristics and prognoses of patients with hepatoid adenocarcinoma and develops an individualized nomogram based on independently associated risk factors to predict overall survival among those with this distinct cancer subtype.

## Introduction

Hepatoid adenocarcinoma of the stomach (HAS) is a unique subtype of gastric cancer (GC), accounting for 0.3% to 1.0% of GC cases.^1^ The pathological diagnosis of HAS is based on histological features similar to those of hepatocellular carcinoma.^2^ Based on the different proportions of hepatoid differentiation areas, cases can be classified as simple HAS, which is defined as the presence of histologically contained hepatoid differentiation areas only, or mixed HAS, which is defined as histologically contained hepatoid differentiation areas plus common adenocarcinoma areas.^3,4^

Given the low incidence of HAS, limited data exist regarding the clinicopathological characteristics and prognoses of patients with this cancer subtype. Most previous studies have reported that patients with HAS have an unfavorable prognosis and a low 5-year survival rate.^1,5,6^ Zhou et al^7^ recently compared the clinicopathological data of 75 patients with HAS and 722 patients with common GC and found differences between the 2 cancer types with regard to tumor invasion depth, human epidermal growth factor receptor levels, human epidermal growth factor receptor 2 expression, antigen Ki-67 levels, preoperative serum carcinoembryonic antigen (CEA) levels (to convert to micrograms per liter, multiply by 1.0), carbohydrate antigen 19-9 levels, and proportion of patients receiving neoadjuvant chemotherapy. The survival analysis after 1:2 propensity matching revealed that 3-year overall survival (OS) rates of patients with HAS compared with common GC were 79.9% vs 96.9%, respectively (log-rank *P* = .20).^7^ However, these studies^1,5,6,7^ were limited by small samples (≤75 participants) and may not accurately reflect the clinicopathological characteristics and prognoses of patients with HAS. An individualized prognostic model has not yet been developed. Therefore, the current study aimed to explore the clinicopathological characteristics and prognoses of those with HAS and to develop and validate an individualized prognostic nomogram to predict OS among patients with HAS from 14 centers in China.

## Methods

This prognostic study was approved by the human ethics review committee of each participating center. All participants provided written informed consent to use and publish their deidentified medical data at the time of their first hospitalization. This study adhered to the Declaration of Helsinki^8^ and followed the Transparent Reporting of a Multivariable Prediction Model for Individual Prognosis or Diagnosis (TRIPOD) reporting guideline

.

### Patients and Definitions

We retrospectively analyzed the clinicopathological data of patients with HAS who were admitted to 14 centers affiliated with the China Hepatoid Adenocarcinoma of the Stomach Study Group between April 1, 2004, and December 31, 2019. Patients were included in the study if they had pathologically confirmed HAS (including both simple and mixed HAS). Patients were excluded if they had remnant GC (n = 5), other cancers (n = 2), and/or an unknown HAS subtype (n = 3). In total, 315 patients with primary HAS were included; of those, 137 patients had simple HAS, and 178 patients had mixed HAS (eTable 1 and eFigure 1 in the Supplement).

For patients without distant metastasis, curative surgery was defined as microscopic tumor-free margin (R0) resection of the primary tumor plus standard lymphadenectomy (limited dissection [D1 or D1-plus] or extended dissection [D2]). For patients with liver metastases, curative surgery was defined as R0 resection of the primary tumor and liver metastases combined with a standard D2 lymphadenectomy^9^; palliative surgery was considered for patients receiving any other type of resection. The resected specimens were carefully examined by 2 experienced pathologists in each center, and tumor staging was performed based on the *AJCC Cancer Staging Manual* (8th edition) tumor-node-metastasis (TNM) staging system for GC from the American Joint Committee on Cancer (AJCC).^10^ For patients with advanced HAS without contraindications, we recommended treatment with 6 to 8 cycles of postoperative adjuvant chemotherapy. Because no international guideline regarding adjuvant chemotherapy for the treatment of those with HAS currently exists, the chemotherapy regimen administered to patients in this study was the same as that used for the treatment of patients with common GC (ie, 5-fluorouracil–based chemotherapy).

### Pathological Diagnosis

Pathological examination is the criterion standard for the diagnosis of HAS, and morphological features are the main basis for pathological diagnosis.^11^ No quantity requirement for the diagnosis of histological hepatoid differentiation was used, and some patients with focal differentiation could have also received a diagnosis of HAS.^4^ Most cases of HAS presented histologically contained hepatoid differentiation areas plus common adenocarcinoma areas, whereas some cases presented only hepatoid differentiation areas.^3,4^ In the areas of hepatoid differentiation, tumor cells were arranged in a trabecular manner or in solid nests separated by narrow fibrous stroma composed of sinusoid-like capillaries. The tumor cells were cuboidal or polygonal with eosinophilic or clear cytoplasm and occasionally contained hyaline globules. The nucleus was large and ovoid and contained 1 to 2 nucleoli.^6^

If histological examination identified only a hepatoid differentiation area, the HAS was defined as simple (eFigure 2 in the Supplement); if both hepatoid differentiation and common adenocarcinoma areas were identified (eFigure 2 in the Supplement), the HAS was defined as mixed. Immunohistochemical staining was helpful for the diagnosis of HAS. More than 90% of patients with HAS expressed α-fetoprotein (AFP) (to convert to micrograms per liter, multiply by 1.0),^1^ which is often positive or highly positive in the hepatoid differentiation area (eFigure 2 in the Supplement), whereas in the common adenocarcinoma area, AFP is generally negative (eFigure 2 in the Supplement). However, a positive AFP result on immunohistochemical staining is not necessary for the diagnosis of HAS.

### Follow-up

The strategy for follow-up of patients with HAS was the same as that used for patients with common GC. Participants were followed up through outpatient visits and telephone calls. Outpatient visits included physical examination, laboratory examination (routine blood test, blood biochemical examination, and measurement of AFP, CEA, and carbohydrate antigen 19-9 levels), chest radiography, abdominal ultrasonography or computed tomography, and annual esophagogastroduodenoscopy examination. All patients were followed up once every 3 to 6 months during the first 2 years, once every 6 to 12 months during the following 3 to 5 years, and once every year thereafter. Overall survival was recorded from the date of surgery or pathological diagnosis to the date of last follow-up or death. Because patients from multiple centers were included in this study, the last follow-up date of each center was different. A median follow-up period of 35.3 months (IQR, 16.0-58.4 months) was calculated using the reverse Kaplan-Meier method.^12,13,14^

### Construction and Validation of Nomogram

Patients were randomly assigned to a derivation cohort (220 patients) and a validation cohort (95 patients) through a random number list generated by SPSS software, version 22.0 (SPSS Statistics). In the derivation cohort, independent prognostic risk factors among patients with HAS were examined using univariable and multivariable Cox mixed-effects models.

Based on these risk factors, we used R software, version 4.0.3 (R Foundation for Statistical Computing), to generate a nomogram to predict OS at 1 and 3 years. Because the current clinical judgment of prognosis among patients with GC is mainly based on TNM stage and the receipt of postoperative adjuvant chemotherapy, we compared the prognostic ability of the nomogram with both the AJCC pathological TNM (pTNM) staging system and a clinical model that included pTNM stage and receipt of adjuvant chemotherapy among the different cohorts.

The performance evaluation of the nomogram included calibration and discrimination. Internal validation was performed using bootstrapping with the 1000 resampling method. Performance was further verified using the Harrell concordance index^15^ and the calibration curve generated by the validation cohort and the whole cohort. In addition, we calculated the Akaike information criterion between the 3 models (derivation, validation, and whole cohorts) to assess the risk of overfitting.^16^ Time-dependent receiver operating characteristic curves were used to compare the dynamic difference of the area under the curve between the 3 models.^17^ A decision curve analysis was performed to determine the clinical usefulness of the nomogram by quantifying the net benefit across a range of risk thresholds.^18^

### Statistical Analysis

Statistical analysis was performed using SPSS statistical software, version 22.0 (SPSS Statistics), and R software, version 4.0.3 (R Foundation for Statistical Computing). Categorical data were summarized as frequencies with percentages, and statistical comparisons for categorical variables were performed using the χ^2^ or Fisher exact test. Continuous variables were reported as means with SDs for variables with normal distribution and medians with IQRs for variables without normal distribution. Between-group means were compared using a *t* test, and medians were compared using the Mann-Whitney *U* test. The Kaplan-Meier method was used to calculate time-specific survival probabilities. A log-rank test was used for statistical comparisons between the survival curves. To account for possible differences in patient characteristics and survival outcomes at different centers, a Cox mixed-effects model^19^ was used to identify independent prognostic factors associated with survival. Variables with *P* < .05 in the univariable analysis were included in the multivariable model. Proportional hazards were evaluated using a proportional hazards assumption test and a Schoenfeld residual test.^20^ All statistical tests were 2-sided with a significance threshold of *P* < .05. Each patient (excluding 5 of 315 patients who received biopsies only) was assigned a risk score based on the nomogram. X-tile software, version 3.6.1 (Rimm Lab, Yale School of Medicine),^21^ was used to examine the diagnostic thresholds for the high-risk and low-risk scores for all patients. Patients with risk scores higher and lower than the diagnostic threshold were classified into high-risk and low-risk groups, respectively.

## Results

### Clinicopathological Characteristics

Among 315 patients with HAS included in the study, the mean (SD) age was 61.9 (10.2) years; 240 patients (76.2%) were male, and 75 patients (23.8%) were female (Table 1). The mean (SD) tumor size was 5.4 (2.6) cm, and the median preoperative serum AFP value was 73.0 ng/mL (IQR, 4.2-798.3 ng/mL). A total of 139 of 223 patients (62.3%) had elevated serum AFP levels (≥20 ng/mL). Among all patients, 117 (37.1%) had gastric antrum tumors, and 34 (10.8%) had preoperative liver metastases. Of the 34 patients with liver metastases, 3 also had distant metastases (2 patients had pulmonary metastases, and 1 patient had pancreatic head metastases). Among 31 patients with synchronous liver metastases only, 26 (83.9%) received curative surgery. Of 315 total patients, 295 (93.7%) received curative surgery, 15 (4.8%) received palliative surgery, and 5 (1.6%) received biopsies. Lymphovascular invasion was noted in 179 patients (56.8%) and perineural invasion in 127 patients (40.3%).

**Table 1.** Demographic, Clinical, Treatment, and Pathological Characteristics of Study Population

Characteristic	No./total No. (%)			*P* value
	All participants with HAS (n = 315)	Participants with simple HAS (n = 137)	Participants with mixed HAS (n = 178)	
**Demographic**				
Age, mean (SD), y	61.9 (10.2)	62.7 (10.5)	61.3 (10.0)	.23
Sex				
Male	240/315 (76.2)	101/137 (73.7)	139/178 (78.1)	.37
Female	75/315 (23.8)	36/137 (26.3)	39/178 (21.9)	
**Clinical**				
BMI, mean (SD)	22.7 (3.0)	22.6 (3.3)	22.7 (2.8)	.82
ASA class				
1	60/315 (19.0)	29/137 (21.2)	31/178 (17.4)	.71
2	211/315 (70.2)	88/137 (64.2)	123/178 (69.1)	
3	35/315 (11.1)	15/137 (10.9)	20/178 (11.2)	
Unknown	9/315 (2.9)	5/137 (3.6)	4/178 (2.2)	
Tumor location				
Upper	110/315 (34.9)	43/137 (31.4)	67/178 (37.6)	.49
Middle	46/315 (14.6)	23/137 (16.8)	23/178 (12.9)	
Lower	117/315 (37.1)	50/137 (36.5)	67/178 (37.6)	
Mixed	42/315 (13.3)	21/137 (15.3)	21/178 (11.8)	
Tumor size, mean (SD), cm	5.4 (2.6)	5.6 (2.8)	5.4 (2.4)	.48
α-Fetoprotein level^a^				
Median (IQR), ng/mL	73.0 (4.2-798.3)	195.9 (8.2-1416.1)	48.9 (3.2-237.3)	**<**.001
≥20 ng/mL	139/223 (62.3)	70/103 (68.0)	69/120 (57.5)	.11
CEA level^a^				
Median (IQR), ng/mL	2.8 (1.7-6.5)	2.7 (1.7-6.5)	3.1 (1.7-7.0)	.49
≥5 ng/mL	98/315 (31.1)	42/137 (30.7)	56/178 (31.5)	.88
CA19-9 level				
Median (IQR), U/mL	9.9 (5.0-19.2)	9.9 (4.8-17.5)	9.9 (5.2-20.0)	.73
≥37 U/mL	35/250 (14.0)	16/112 (14.3)	19/138 (13.8)	.91
**Treatment**				
Type of resection				
Gastrectomy				.06
Total	155/315 (49.2)	66/137 (48.2)	89/178 (50.0)	
Distal	111/315 (35.2)	45/137 (32.8)	66/178 (37.1)	
Proximal	29/315 (9.2)	11/137 (8.0)	18/178 (10.1)	
Palliative surgery	15/315 (4.8)	11/137 (8.0)	4/178 (2.2)	
Biopsy only	5/315 (1.6)	4/137 (2.9)	1/178 (0.6)	
Neoadjuvant chemotherapy				
Yes	21/315 (6.7)	11/137 (8.0)	10/178 (5.6)	.40
No	294/315 (93.3)	126/137 (92.0)	168/178 (94.4)	
Adjuvant chemotherapy				
Yes	152/315 (48.3)	72/137 (52.6)	80/178 (44.9)	.18
No	163/315 (51.7)	65/137 (47.4)	98/178 (55.1)	
**Pathological**				
Lymph nodes, mean (SD)				
Examined	30.8 (14.5)	30.1 (14.8)	31.4 (14.3)	.43
Metastatic	6.8 (8.2)	6.1 (6.9)	7.4 (9.1)	.15
Lymphovascular invasion				
Absent	131/315 (41.6)	58/137 (42.3)	73/178 (41.0)	.23
Present	179/315 (56.8)	75/137 (54.7)	104/178 (58.4)	
Unknown	5/315 (1.6)	4/137 (2.9)	1/178 (0.6)	
Perineural invasion				
Absent	183/315 (58.1)	82/137 (59.9)	101/178 (56.7)	.18
Present	127/315 (40.3)	51/137 (37.2)	76/178 (42.7)	
Unknown	5/315 (1.6)	4/137 (2.9)	1/178 (0.6)	
pT category^b^				
T1	24/315 (7.6)	9/137 (6.6)	15/178 (8.4)	.23
T2	38/315 (12.1)	16/137 (11.7)	22/178 (12.4)	
T3	85/315 (27.0)	30/137 (21.9)	55/178 (30.9)	
T4a	140/315 (44.4)	66/137 (48.2)	74/178 (41.6)	
T4b	23/315 (7.3)	12/137 (8.8)	11/178 (6.2)	
Unknown	5/315 (1.6)	4/137 (2.9)	1/178 (0.6)	
pN category^b^				
N0	57/315 (18.1)	27/137 (19.7)	30/178 (16.9)	.12
N1	61/315 (19.4)	22/137 (16.1)	39/178 (21.9)	
N2	83/315 (26.3)	43/137 (31.4)	40/178 (22.5)	
N3a	70/315 (22.2)	28/137 (20.4)	42/178 (23.6)	
N3b	39/315 (12.4)	12/137 (8.8)	26/178 (14.6)	
Unknown	5/315 (1.6)	4/137 (2.9)	1/178 (0.6)	
pTNM stage^b^				
IA	13/315 (4.1)	4/137 (2.9)	9/178 (5.1)	.004
IB	21/315 (6.7)	10/137 (7.3)	11/178 (6.2)	
IIA	21/315 (6.7)	6/137 (4.4)	15/178 (8.4)	
IIB	38/315 (12.1)	18/137 (13.1)	20/178 (11.2)	
IIIA	84/315 (26.7)	35/137 (25.5)	49/178 (27.5)	
IIIB	58/315 (18.4)	21/137 (15.3)	37/178 (20.8)	
IIIC	33/315 (10.5)	10/137 (7.3)	23/178 (12.9)	
IV	46/315 (14.6)	33/137 (24.1)	13/178 (7.3)	
Unknown	1/315 (0.3)	0	1/178 (0.6)	
Liver metastasis				
Yes	34/315 (10.8)	23/137 (16.8)	11/178 (6.2)	.003
No	281/315 (89.2)	114/137 (83.2)	167/178 (93.8)	

Abbreviations: ASA, American Society of Anesthesiologists; BMI, body mass index (calculated as weight in kilograms divided by height in meters squared); CA19-9, carbohydrate antigen 19-9; CEA, carcinoembryonic antigen; HAS, hepatoid adenocarcinoma of the stomach; pN, pathological node; pT, pathological tumor; pTNM, pathological tumor-node-metastasis.

^a^ To convert to micrograms per liter, multiply by 1.0.

^b^ Based on the *AJCC Cancer Staging Manual* (8th edition) staging system.

Based on the AJCC pTNM staging system, 24 patients (7.6%) had early HAS (pathological tumor category 1 [pT1]), and 286 patients (90.8%) had advanced HAS (pT2-pT4), with 140 patients (44.4%) having pT4a and 23 patients (7.3%) having pT4b HAS. A total of 57 patients (18.1%) had pathological node-negative (pN0) cancer, and 253 patients (80.3%) had pathological node-positive (pN1-pN3) cancer. Comparison of the clinicopathological data of patients with simple vs mixed HAS revealed that preoperative AFP levels in the simple HAS group were higher than those of the mixed HAS group (median, 195.9 ng/mL [IQR, 8.2-1416.1 ng/mL] vs 48.9 ng/mL [IQR, 3.2-237.3 ng/mL], respectively; *P* < .001), and those with simple HAS vs mixed HAS had a higher rate of preoperative liver metastasis (23 of 137 patients [16.8%] vs 11 of 178 patients [6.2%]; *P* = .003). A total of 17 of 21 patients (81.0%) with simple HAS and synchronous liver metastasis received curative surgery. Patients with simple vs mixed HAS also had different pTNM staging, particularly regarding later stages of disease (eg, 33 of 137 patients [24.1%] with simple HAS vs 13 of 178 patients [7.3%] with mixed HAS had stage IV cancer; *P* = .004).

### AJCC TNM Staging Survival Analysis

**Figure 1.**
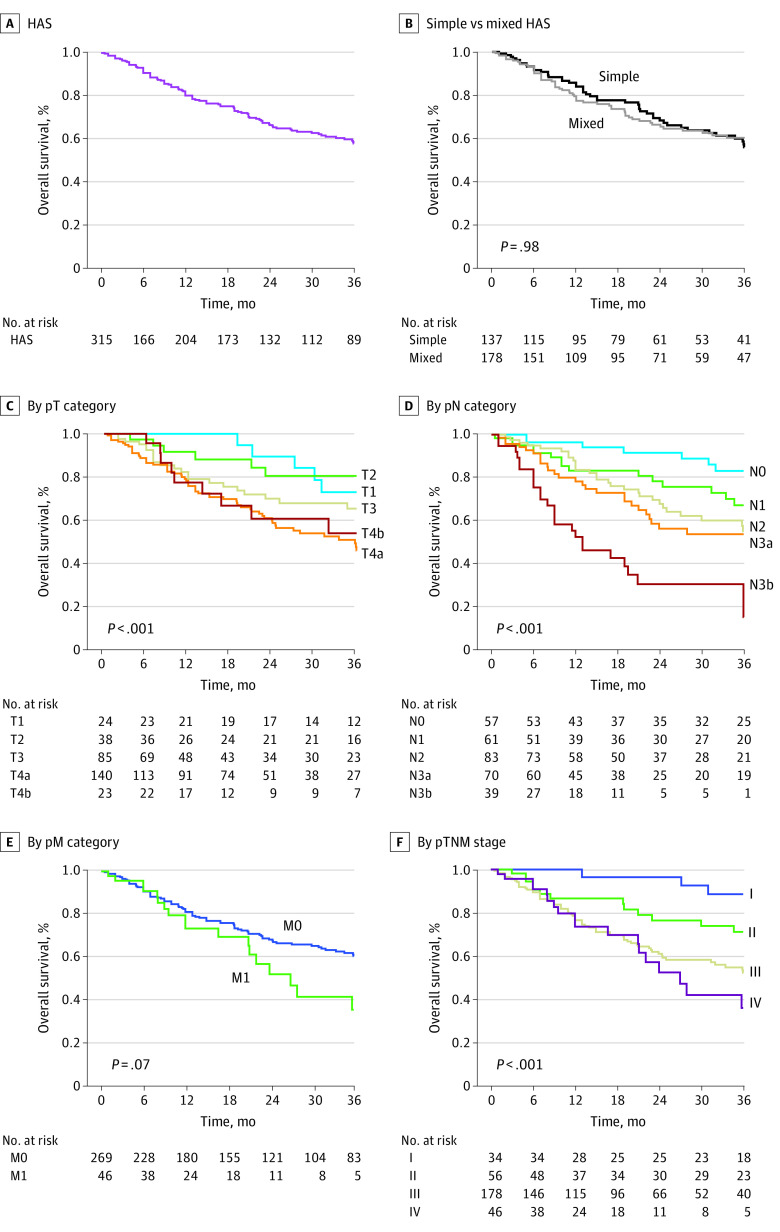
Overall Survival Among Patients With Hepatoid Adenocarcinoma of the Stomach (HAS) pM indicates pathological metastasis; pN, pathological node; pT, pathological tumor; and pTNM, pathological tumor-node-metastasis.

The 3-year OS rate in the total sample of 315 patients with HAS was 58.1% (Figure 1). No significant difference in the 3-year OS rate was found between the simple and mixed HAS groups (56.0% vs 60.0%, respectively; log-rank *P* = .98). Based on pT category, the 3-year OS rates were 73.0% for those with T1 cancer, 80.5% for those with T2 cancer, 65.3% for those with T3 cancer, 46.0% for those with T4a cancer, and 53.9% for those with T4b cancer (log-rank *P* < .001). Based on pN category, the 3-year OS rates were 83.1% for those with N0 cancer, 67.2% for those with N1 cancer, 55.1% for those with N2 cancer, 53.9% for those with N3a cancer, and 15.3% for those with N3b cancer (log-rank *P* < .001). Based on pathological metastasis (pM) category, the 3-year OS rates were 60.8% for those with M0 cancer and 35.9% for those with M1 cancer, which was not a statistically significant survival difference (log-rank *P* = .07). Based on AJCC pTNM stage, the 3-year OS rates were 88.5% for those with stage I cancer, 71.1% for those with stage II cancer, 52.1% for those with stage III cancer, and 35.9% for those with stage IV cancer (log-rank *P* < .001).

### Prognostic Risk Factors and Nomogram Construction

**Figure 2.**
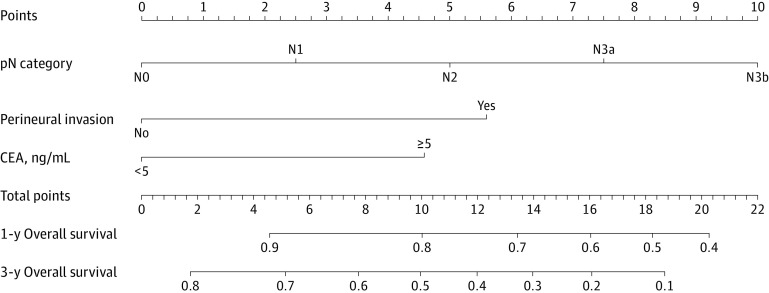
Nomogram Based on Results of Multivariable Cox Analysis of Derivation Cohort Points were assigned for pathological node category, perineural invasion, and carcinoembryonic antigen by drawing a line upward from the corresponding values to the Points line. The sum of these 3 points, plotted on the Total Points line, corresponds to estimates of the overall survival of patients with hepatoid adenocarcinoma of the stomach at 1 year and 3 years. CEA indicates carcinoembryonic antigen (to convert to micrograms per liter, multiply by 1.0); and pN, pathological node.

To evaluate the nomogram, the total sample of 315 patients with HAS was randomly divided into a derivation cohort (220 patients [69.8%]) and a validation cohort (95 patients [30.2%]). The derivation and validation cohorts were balanced and comparable in terms of clinicopathological data (eg, mean [SD] age, 62.0 [10.5] years vs 61.7 [9.6] years; 168 men [76.4%] vs 72 men [75.8%]; 128 patients [58.2%] vs 51 patients [53.7%] with lymphovascular invasion; 88 patients [40.0%] vs 39 patients [41.1%] with perineural invasion, respectively) (eTable 2 in the Supplement). In the derivation cohort, the univariable Cox analysis revealed that the presence of lymphovascular invasion (hazard ratio [HR], 2.79; 95% CI, 1.64-4.75; *P* < .001), the presence of perineural invasion (HR, 2.88; 95% CI, 1.81-4.60; *P* < .001), a preoperative CEA level of 5 ng/mL or greater (HR, 2.33; 95% CI, 1.50-3.64; *P* < .001), 1 pT category (pT4a: HR, 3.57; 95% CI, 1.09-11.72; *P* = .04), and 3 pN categories (pN2: HR, 2.62 [95% CI, 1.12-6.15; *P* = .03]; pN3a: 3.46 [95% CI, 1.46-8.21; *P* = .005]; pN3b: HR, 7.78 [95% CI, 3.21-18.90; *P* < .001]) were all associated with OS among patients with HAS. The multivariable Cox analysis found that the presence of perineural invasion (HR, 2.13; 95% CI, 1.27-3.55; *P* = .009), a preoperative CEA level of 5 ng/mL or greater (HR, 1.72; 95% CI, 1.08-2.74; *P* = .03), and 1 pN category (pN3b: HR, 3.72; 95% CI, 1.34-10.32; *P* = .01) were independently associated with OS (eTable 3 in the Supplement). The variables associated with OS in the multivariable analysis were incorporated into the prognostic model to develop an individualized nomogram of OS at 1 and 3 years (Figure 2).

### Nomogram Validation

The calibration curve of the nomogram model had good consistency in each cohort (eFigure 3 in the Supplement). Among all cohorts, the concordance indices of the nomogram (derivation cohort: 0.72 [95% CI, 0.66-0.78]; validation cohort: 0.72 [95% CI, 0.63-0.81]; whole cohort: 0.71 [95% CI, 0.66-0.76]) were higher than those of the AJCC pTNM staging system (derivation cohort: 0.63 [95% CI, 0.57-0.69]; validation cohort: 0.65 [95% CI, 0.56-0.75]; whole cohort: 0.64 [95% CI, 0.59-0.69]) and the clinical model (derivation cohort: 0.64 [95% CI, 0.58-0.69]; validation cohort: 0.65 [95% CI, 0.56-0.75]; whole cohort: 0.64 [95% CI, 0.59-0.69]) (Table 2). The difference in the concordance index of the nomogram in the derivation and whole cohorts was significantly higher than that of the AJCC pTNM staging system (derivation cohort: 0.09 [95% CI, 0.03-0.14; *P* = .004]; whole cohort: 0.08 [95% CI, 0.04-0.11; *P* < .001]) and the clinical model (derivation cohort: 0.08 [95% CI, 0.02-0.14; *P* = .009]; whole cohort: 0.07 [95% CI, 0.03-0.11; *P* < .001]). The nomogram had a lower Akaike information criterion score than both the AJCC pTNM staging system and the clinical model, independent of cohort (Table 2). For example, among the whole cohort, the Akaike information criterion score was 1071.65 for the nomogram, 1110.69 for the AJCC staging system, and 1112.53 for the clinical model.

**Table 2.** Concordance Index and Akaike Information Criterion for Nomogram, pTNM Staging, and Clinical Models by Cohort

Cohort	Concordance index (95% CI)	Change in concordance index (95% CI)	Akaike information criterion	*P* value
Derivation				
Nomogram	0.72 (0.66 to 0.78)	NA	728.43	NA
pTNM staging^a^	0.63 (0.57 to 0.69)	0.09 (0.03 to 0.14)	758.72	.004
Clinical model^b^	0.64 (0.58 to 0.69)	0.08 (0.02 to 0.14)	760.54	.009
Validation				
Nomogram	0.72 (0.63 to 0.81)	NA	222.76	NA
pTNM staging^a^	0.65 (0.56 to 0.75)	0.07 (−0.02 to 0.13)	227.38	.08
Clinical model^b^	0.65 (0.56 to 0.75)	0.07 (−0.01 to 0.15)	229.38	.12
Whole				
Nomogram	0.71 (0.66 to 0.76)	NA	1071.65	NA
pTNM staging^a^	0.64 (0.59 to 0.69)	0.08 (0.04 to 0.11)	1110.69	<.001
Clinical model^b^	0.64 (0.59 to 0.69)	0.07 (0.03 to 0.11)	1112.53	<.001

Abbreviations: NA, not applicable; pTNM, pathological tumor-node-metastasis.

^a^ Based on the American Joint Committee on Cancer’s *AJCC Cancer Staging Manual* (8th edition) staging system.

^b^ Clinical model included pTNM stage and receipt of adjuvant chemotherapy.

The time-dependent receiver operating characteristic curve found that the nomogram predicted OS at each postoperative point better than the AJCC pTNM staging system and the clinical model, independent of cohort (eFigure 4 in the Supplement). A decision curve analysis evaluating the clinical usefulness of each model revealed that the nomogram had a higher net benefit than the other 2 models with the same risk thresholds, independent of cohort (eFigure 5 in the Supplement).

We also performed a sensitivity analysis in which 79 patients from 1 center (Fujian Medical University Union Hospital) were assigned to the validation cohort and 236 patients from the other 13 centers were assigned to the derivation cohort. The results revealed that the concordance indices of the nomogram among the derivation and validation cohorts (0.71 [95% CI, 0.65-0.77] and 0.74 [95% CI, 0.62-0.85], respectively) were higher than those of the AJCC pTNM staging system (0.63 [95% CI, 0.57-0.68] and 0.69 [95% CI, 0.57-0.81]) and the clinical model (0.62 [95% CI, 0.57-0.68] and 0.69 [95% CI, 0.57-0.81]). The nomogram had a lower Akaike information criterion score in both the derivation and validation cohorts (818.06 and 149.99, respectively) than the AJCC pTNM staging system (841.72 and 156.80) and the clinical model (843.72 and 158.80) (eTable 4 in the Supplement). An additional stratified analysis was performed based on different histological types, and the calibration curve of the nomogram model revealed good consistency between the simple and mixed HAS groups; the prognostic prediction performance of the nomogram was also better than that of the other 2 models in both the simple and mixed HAS groups (eTable 5 and eFigures 6-8 in the Supplement).

### Risk Grouping and Nomogram Survival Analysis

After exclusion of 5 patients who received biopsies only, and without knowledge of patient pN category and perineural invasion status, we used the nomogram to assign risk scores to 310 patients, with scores ranging from 0 to 21 points. Based on the 3-year OS rate, the minimum *P* value of the log-rank χ^2^ test and cutoff values of the high-risk and low-risk groups were calculated using X-tile software. When the risk score was 10 points, the corresponding χ^2^ value was 49.23 (*P* < .001). Based on this cutoff value, patients with a risk score higher than 10 points were defined as high risk, and patients with a risk score lower than 10 points were defined as low risk (eFigure 9 in the Supplement). Compared with the tumors of patients in the low-risk group, those of patients in the high-risk group were larger and more often located in the upper stomach; had higher preoperative CEA levels, higher rates of total gastrectomy, and a larger number of metastatic lymph nodes; commonly presented with lymphovascular and perineural invasion; and were classified in a more advanced postoperative pathological stage (eTable 6 in the Supplement).

**Figure 3.**
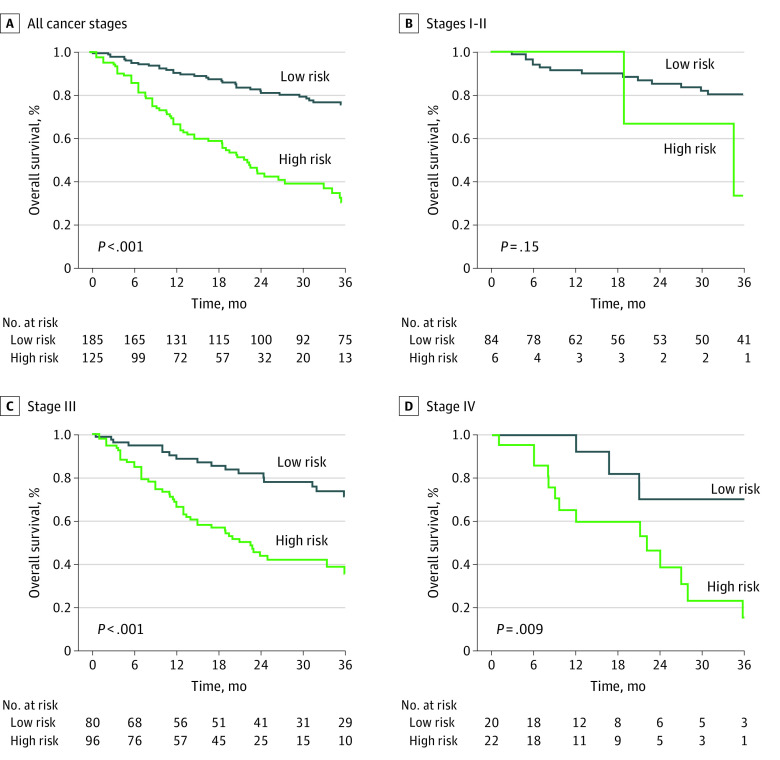
Overall Survival of Low-Risk vs High-Risk Groups B, *P* value derived from exact log-rank test.

The 3-year OS rate of patients in the high-risk group was 29.7%, which was significantly lower than the 75.9% OS rate found among low-risk patients (*P* < .001) (Figure 3). An additional stratified analysis based on AJCC pTNM stage revealed that the 3-year OS rate of high-risk and low-risk patients could be distinguished as stage I to II (33.3% vs 80.2%, respectively; exact log-rank *P* = .15), stage III (34.3% vs 71.3%; log-rank *P* < .001), and stage IV (15.5% vs 70.3%; log-rank *P* = .009) (Figure 3). A stratified survival analysis based on histological type found that regardless of simple or mixed HAS status, the 3-year OS rate of patients in the high-risk group was significantly lower than that of the low-risk group (simple HAS: 21.0% vs 73.7%, respectively; log-rank *P* < .001; mixed HAS: 34.8% vs 77.9%; log-rank *P* < .001) (eFigure 10 in the Supplement).

## Discussion

This prognostic study examined the clinicopathological characteristics and prognoses of patients with HAS, a distinct type of cancer with a low incidence. The general characteristics of HAS were first identified by Ishikura et al^22^ in 1985, and studies of HAS have been performed since then.^23,24^ Hepatoid adenocarcinoma of the stomach occurs more often in middle-aged and older men, is more commonly located in the gastric antrum, presents varying levels of elevated serum AFP, and is more likely to be accompanied by lymph node and liver metastasis.^4,25,26^ The mean (SD) age in our sample of 315 patients with HAS was 61.9 (10.2) years, the male to female ratio was approximately 3:1, gastric antrum tumors accounted for 37.1% of cases, and 81.6% of patients had lymph node metastases, consistent with previous reports.^4,25,26^ In addition, to our knowledge, this study was the first to compare the clinicopathological characteristics of patients with simple vs mixed HAS, finding that the 2 groups had similarities in most clinicopathological characteristics; however, patients with simple HAS had higher preoperative serum AFP levels and a higher rate of liver metastasis compared with those with mixed HAS. These findings suggest that monitoring of serum AFP levels and the performance of liver imaging may be more important for patients with simple HAS.

Previous studies have found that, among patients with HAS, the 1-year survival rate was 30% to 44%, the 3-year survival rate was 13% to 23%, and the 5-year survival rate was 9% to 20%.^3,24,27,28^ The 3-year OS rate in the current study was 58.1%, which is higher than the rates reported in most of those previous studies and may be associated with the fact that most patients in the current study received curative surgery. Similar to a previous study,^3^ we did not find a difference in OS between those with simple vs mixed HAS, suggesting that the hepatoid differentiation area proportion may not be an independent risk factor associated with HAS prognosis. Although patients with simple HAS had more liver metastases, 17 of 21 patients (81.0%) with simple HAS and synchronous liver metastasis in this study received curative surgery, suggesting that among those with HAS and liver metastasis only, the receipt of radical resection of primary and metastatic lesions may still improve the prognosis; however, further studies with larger samples and longer follow-up periods are needed to confirm this finding. In addition, although surgical resection of liver metastases in patients with GC has not been explicitly recommended by guidelines, a systematic review reported long-term survival benefits of surgical resection of liver metastases among those with GC.^29^ Therefore, it is likely that when liver metastases meet the technical requirements for resectability and when hepatectomy can be tolerated (as determined by liver reserve function assessment), patients with HAS and liver metastasis can be treated with R0 resection of the primary tumor and liver metastases combined with a standard D2 lymphadenectomy, which can achieve the purpose of curative surgery. Because of the lack of high-level evidence, among patients with HAS and other distant metastases, the best treatment options may be chemotherapy, radiotherapy, palliative care, or best supportive care, as recommended in current treatment guidelines for patients with GC.^30,31^

Although most HAS is accompanied by an increase in preoperative serum AFP levels, we found that elevated AFP (≥20 ng/mL) was not associated with prognosis among those with HAS, which is consistent with previous results^3,27^ and suggests that the diagnostic value of preoperative AFP level among patients with HAS may be greater than its prognostic value. However, because of the lack of data on postoperative serum AFP levels, we were unable to examine the association of postoperative AFP levels and the changes between preoperative and postoperative AFP levels with the prognosis of patients with HAS. In addition, we found no significant difference in survival between patients who did and did not receive adjuvant chemotherapy. Kamoshida et al^32^ found that patients with HAS lacked thymidine phosphorylase, which has an important role in the activation of fluorouracil but is high in dihydropyrimidine dehydrogenase, which is responsible for degrading fluorouracil. This factor may be one of the reasons for the insufficient response of HAS to traditional chemotherapy regimens. Therefore, further assessment of appropriate adjuvant treatment regimens for patients with HAS is warranted.

Previous studies have reported that, among patients with advanced GC, the presence of perineural invasion is associated with local recurrence and worse prognosis.^33^ Perineural invasion has been found to be an independent factor associated with GC metastasis in addition to direct invasion, lymphatic metastasis, hematological metastasis, and peritoneal metastasis.^34,35^ Our results also revealed that perineural invasion was an independent risk factor associated with worse prognosis in patients with HAS. In addition, an elevated preoperative serum CEA level has been reported to be independently associated with the prognosis of various cancers.^36,37,38,39^ We found that a preoperative CEA level of 5 ng/mL or greater was also independently associated with HAS. The prognosis of patients with elevated CEA levels was significantly worse than that of patients with normal CEA levels; therefore, clinical attention to CEA levels is warranted.

To better predict the long-term prognosis of patients with HAS and develop targeted treatment and follow-up strategies, we developed an individualized nomogram based on 3 factors independently associated with prognosis (perineural invasion, preoperative CEA level, and pN category 3b). To our knowledge, the current study is the first to use a nomogram to predict the OS of patients with HAS. The nomogram had good performance in the derivation, validation, and whole cohorts and was suitable for patients with both simple and mixed HAS. Furthermore, according to the risk scores generated by the nomogram for the classification of high-risk and low-risk patients, there was a significant difference in survival between the 2 groups, independent of histological classification. The prognosis of high-risk and low-risk patients could be further distinguished, especially in patients with stage III and IV cancer, suggesting that the nomogram developed in this study may be used as a supplement to the current AJCC TNM staging system.

### Limitations

This study has several limitations. It is a multicenter study from China, with a relatively small number of patients with HAS in each center, and the results lack verification of data from Western populations. Nevertheless, the nomogram we developed is based on simple parameters that are easy to use. With the improvement of pathological diagnosis and the increase in clinicians’ understanding of HAS, we hope that, in the future, we can replicate our findings in centers with large samples and include data from Western countries to verify the applicability of the nomogram. In addition, this study uses a retrospective design, and the number of patients with HAS in each center was unevenly distributed; therefore, bias was inevitable. Moreover, to conduct a study among a large sample of patients with HAS, we retrospectively collected data from 14 centers. However, it is difficult for us to obtain complete data on patients with common GC, who comprise a substantially larger population than those with HAS. Therefore, we cannot compare the clinicopathological characteristics and prognoses of those with HAS vs common GC. Nevertheless, the main purpose of this study was to develop an individualized prognostic model for HAS and perform a comparative study of clinicopathological characteristics and prognoses among patients with simple vs mixed HAS, which has not, to our knowledge, been previously investigated.

## Conclusions

This study combined HAS data from 14 centers in China to explore the clinicopathological characteristics and prognostic risk factors of patients with HAS. Consistent with previous studies, this study found that the preoperative serum AFP levels of those with HAS were elevated and that HAS easily metastasized to the liver. In addition, the current study compared patients with simple vs mixed HAS for the first time to date, finding that those with simple HAS had a significantly higher level of serum AFP and a higher rate of liver metastases than those with mixed HAS; however, the long-term prognoses of patients with the 2 types of HAS were similar. This study also developed an individualized nomogram based on risk factors that were independently associated with worse prognosis (perineural invasion, preoperative CEA levels of 5 ng/mL or greater, and pN category 3b); the nomogram had good performance in predicting OS among patients with HAS and may be useful as a supplement to the current AJCC TNM staging system.

## Notes

Supplement. **eTable 1.** Number of Patients With HAS at Each Center **eTable 2.** Demographic and Clinicopathological Characteristics of Derivation and Validation Cohorts **eTable 3.** Univariable and Multivariable Analyses of Risk Factors Associated With Overall Survival in Derivation Cohort Using Cox Mixed-Effects Model **eTable 4.** Comparison of Concordance Index and Akaike Information Criterion of Nomogram, AJCC pTNM Staging System, and Clinical Model Between Validation Cohort (FMUUH) and Derivation Cohort (Other 13 Centers) **eTable 5.** Comparison of Concordance Index and Akaike Information Criterion of Nomogram, AJCC pTNM Staging System, and Clinical Model Between Simple and Mixed HAS Groups **eTable 6.** Demographic and Clinicopathological Characteristics of Low-Risk and High-Risk Groups **eFigure 1.** Study Flow Diagram **eFigure 2.** Histopathological Components of HAS **eFigure 3.** Calibration Curves for Overall Survival Nomogram Models in Derivation, Validation, and Whole Cohorts **eFigure 4**. Time-Dependent ROC Curve Comparing AUCs of Nomogram, AJCC pTNM Staging System, and Clinical Model in Derivation, Validation, and Whole Cohorts for Overall Survival of HAS **eFigure 5.** Decision Curve Analysis of Each Model for Estimating 3-Year Overall Survival in Derivation, Validation, and Whole Cohorts **eFigure 6.** Calibration Curves for Overall Survival Nomogram Models in Simple and Mixed HAS Groups **eFigure 7.** Time-Dependent ROC Curve Comparing AUCs of Nomogram, AJCC pTNM Staging System, and Clinical Model in Simple and Mixed HAS Groups for Overall Survival **eFigure 8.** Decision Curve Analysis of Each Model for Estimating 3-Year Overall Survival in Simple and Mixed HAS Groups **eFigure 9.** Division of Patients by Cutoff Points Produced by X-Tile Plot **eFigure 10.** Kaplan-Meier Analysis of Overall Survival Between Low-Risk and High-Risk Groups by Histological Type
