# Supplementary material for: Assessment of Clinicopathological Characteristics and Development of an Individualized Prognostic Model for Patients With Hepatoid Adenocarcinoma of the Stomach
Source: JAMA Netw Open. 2021 Oct 5;4(10):e2128217. doi: 10.1001/jamanetworkopen.2021.28217 (PMC8493440; doi:10.1001/jamanetworkopen.2021.28217)
Supplement: Supplement. — eTable 1. Number of Patients With HAS at Each Center eTable 2. Demographic and Clinicopathological Characteristics of Derivation and Validation Cohorts eTable 3. Univariable and Multivariable Analyses of Risk Factors Associated With Overall Survival in Derivation Cohort Using Cox Mixed-Effects Model eTable 4. Comparison of Concordance Index and Akaike Information Criterion of Nomogram, AJCC pTNM Staging System, and Clinical Model Between Validation Cohort (FMUUH) and Derivation Cohort (Other 13 Centers) eTable 5. Comparison of Concordance Index and Akaike Information Criterion of Nomogram, AJCC pTNM Staging System, and Clinical Model Between Simple and Mixed HAS Groups eTable 6. Demographic and Clinicopathological Characteristics of Low-Risk and High-Risk Groups eFigure 1. Study Flow Diagram eFigure 2. Histopathological Components of HAS eFigure 3. Calibration Curves for Overall Survival Nomogram Models in Derivation, Validation, and Whole Cohorts eFigure 4. Time-Dependent ROC Curve Comparing AUCs of Nomogram, AJCC pTNM Staging System, and Clinical Model in Derivation, Validation, and Whole Cohorts for Overall Survival of HAS eFigure 5. Decision Curve Analysis of Each Model for Estimating 3-Year Overall Survival in Derivation, Validation, and Whole Cohorts eFigure 6. Calibration Curves for Overall Survival Nomogram Models in Simple and Mixed HAS Groups eFigure 7. Time-Dependent ROC Curve Comparing AUCs of Nomogram, AJCC pTNM Staging System, and Clinical Model in Simple and Mixed HAS Groups for Overall Survival eFigure 8. Decision Curve Analysis of Each Model for Estimating 3-Year Overall Survival in Simple and Mixed HAS Groups eFigure 9. Division of Patients by Cutoff Points Produced by X-Tile Plot eFigure 10. Kaplan-Meier Analysis of Overall Survival Between Low-Risk and High-Risk Groups by Histological Type [file jamanetwopen-e2128217-s001.pdf]

Supplementary Online Content

Lin JX, Wang ZK, Hong QQ, et al. Assessment of clinicopathological characteristics and development of an individualized prognostic model for patients with hepatoid adenocarcinoma of the stomach. *JAMA Netw Open*. 2021;4(10):e2128217. doi:10.1001/jamanetworkopen.2021.28217

eTable 1. Number of Patients With HAS at Each Center

eTable 2. Demographic and Clinicopathological Characteristics of Derivation and Validation Cohorts

eTable 3. Univariable and Multivariable Analyses of Risk Factors Associated With Overall Survival in Derivation Cohort Using Cox Mixed-Effects Model

eTable 4. Comparison of Concordance Index and Akaike Information Criterion of Nomogram, AJCC pTNM Staging System, and Clinical Model Between Valication Cohort (FMUUH) and Derivation Cohort (Other 13 Centers)

eTable 5. Comparison of Concordance Index and Akaike Information Criterion of Nomogram, AJCC pTNM Staging System, and Clinical Model Between Simple and Mixed HAS Groups

eTable 6. Demographic and Clinicopathological Characteristics of Low-Risk and High-Risk Groups

eFigure 1. Study Flow Diagram

eFigure 2. Histopathological Components of HAS

eFigure 3. Calibration Curves for Overall Survival Nomogram Models in Derivation, Validation, and Whole Cohorts

eFigure 4. Time-Dependent ROC Curve Comparing AUCs of Nomogram, AJCC pTNM Staging System, and Clinical Model in Derivation, Validation, and Whole Cohorts for Overall Survival of HAS

eFigure 5. Decision Curve Analysis of Each Model for Estimating 3-Year Overall Survival in Derivation, Validation, and Whole Cohorts

eFigure 6. Calibration Curves for Overall Survival Nomogram Models in Simple and Mixed HAS Groups

eFigure 7. Time-Dependent ROC Curve Comparing AUCs of Nomogram, AJCC pTNM Staging System, and Clinical Model in Simple and Mixed HAS Groups for Overall Survival

eFigure 8. Decision Curve Analysis of Each Model for Estimating 3-Year Overall Survival in Simple and Mixed HAS Groups

eFigure 9. Division of Patients by Cutoff Points Produced by X-Tile Plot

© 2021 Lin JX et al. *JAMA Network Open*.

eFigure 10. Kaplan-Meier Analysis of Overall Survival Between Low-Risk and High-Risk Groups by Histological Type

This supplementary material has been provided by the authors to give readers additional information about their work.

© 2021 Lin JX et al. *JAMA Network Open*.

eTable 1. Number of Patients With HAS at Each Center

Centers	No. of patients
Fujian Medical University Union Hospital	79
the First Affiliated Hospital of Xiamen University	58
Union Hospital affiliated to Tongji Medical College of Huazhong University of Science and Technology	34
Renji Hospital Affiliated to Shanghai Jiaotong University School of Medicine	31
the First Hospital of Jilin University	30
Shandong Provincial Hospital	22
Jiangsu Province Hospital Affiliated to Nanjing Medical University	15
The Second Affiliated Hospital of Fujian Medical University	14
The Affiliated Hospital of Putian City	9
The Sixth Affiliated Hospital of Sun Yat-sen University	9
The First Hospital of Lanzhou University	6
Xiamen University Affiliated Zhongshan Hospital	5
Quanzhou First Hospital Affiliated to Fujian Medical University	2
Longyan First Hospital Affiliated to Fujian Medical University	1

Abbreviations: HAS, hepatoid adenocarcinoma of the stomach.

© 2021 Lin JX et al. *JAMA Network Open*.

eTable 2. Demographic and Clinicopathological Characteristics of Derivation and Validation Cohorts

Characteristics	Derivation cohort (n=220)	Validation cohort (n=95) N (%)	P
**Clinical Factors**			
Age, yr, mean±SD	62.0±10.5	61.7±9.6	.79
BMI, kg/m^2, mean±SD	22.7±3.1	22.6±2.7	.83
Sex			.91
Male	168(76.4)	72(75.8)	
Female	52(23.6)	23(24.2)	
ASA scores			.70
I	42(19.1)	18(18.9)	
II	144(65.5)	67(70.5)	
III	27(12.3)	8(8.4)	
Unknown	7 (3.2)	2 (2.1)	
Tumor locations			.46
Upper	75(34.1)	35(36.8)	
Middle	36(16.4)	10(10.5)	
Lower	78(35.5)	39(41.1)	
Mix	31(14.1)	11(11.6)	
Tumor size, cm, mean±SD	5.4±2.5	5.6±2.7	.52
AFP, ng/mL, median (IQR) [#]	64.8 (3.7-733.1)	107.5 (8.2-894.1)	.33
CEA, ng/mL, median (IQR)	2.7 (1.7-6.2)	3.8 (2.1-7.3)	.11
CA19-9, U/mL, median (IQR) [#]	9.6 (4.8-19.3)	10.7 (5.3-19.6)	.53
**Treatment Factors**			
Type of resection			.44
Total gastrectomy	110(50.0)	45(47.4)	
Distal gastrectomy	74(33.6)	37(38.9)	
Proximal gastrectomy	19(8.6)	10(10.5)	
Palliative surgery	12(5.5)	3(3.2)	
Biopsy only	5(2.3)	0 (0)	
Neoadjuvant chemotherapy			.51
Yes	16(7.3)	5(5.3)	
No	204(92.7)	90(94.7)	
Adjuvant chemotherapy			.84
Yes	107(48.6)	45(47.4)	
No	113(51.4)	50(52.6)	
**Pathological Factors**			
Examined lymph nodes, mean±SD	30.9±13.9	30.5±15.7	.83
Metastatic lymph nodes,	7.2±8.9	6.0±6.6	.22
Lymphovascular invasion			.21
Absent	87(39.5)	44(46.3)	
Present	128(58.2)	51(53.7)	

© 2021 Lin JX et al. *JAMA Network Open.*

Unknown	5 (2.3)	0	
Perineural invasion			.33
Absent	127(57.7)	56(58.9)	
Present	88(40.0)	39(41.1)	
Unknown	5 (2.3)	0	
pT category			.70
T1	15(6.8)	9(9.5)	
T2	26(11.8)	12(12.6)	
T3	61(27.7)	24(25.3)	
T4a	97(44.1)	43(45.3)	
T4b	16(7.3)	7(7.4)	
Unknown	5 (2.3)	0	
pN category			.55
N0	40(18.2)	17(17.9)	
N1	39(17.7)	22(23.2)	
N2	60(27.3)	23(24.2)	
N3a	47(21.4)	23(24.2)	
N3b	29(13.2)	10(10.5)	
Unknown	5 (2.3)	0	
pTNM stage			.91
IA	8 (3.6)	5 (5.3)	
IB	16 (7.3)	5 (5.3)	
IIA	14 (6.4)	7 (7.4)	
IIB	27 (12.3)	11 (11.6)	
IIIA	54 (24.5)	30 (31.6)	
IIIB	43 (19.5)	15 (15.8)	
IIIC	24 (10.9)	9 (9.5)	
IV	33 (15.0)	13 (13.7)	
Unknown	1 (0.5)	0	
Liver metastasis			.62
Yes	25 (11.4)	9 (9.5)	
No	195 (88.6)	86 (90.5)	
Histological type			.12
SHAS	102 (46.4)	35 (36.8)	
MHAS	118 (53.6)	60 (63.2)	

#Ninety-two AFP and 65 CA19-9 were not recorded.

Abbreviations: SD, standard deviation; BMI, body mass index; ASA: American Society of Anesthesiologists; AFP, alpha-fetoprotein; CEA, carcinoembryonic antigen; CA19-9, carbohydrate antigen 19-9; IQR, interquartile range; pT, pathological tumor; pN, pathological node; pM, pathological metastasis; pTNM, pathological tumor-node-metastasis; SHAS, simple hepatoid adenocarcinoma of the stomach; MHAS, mixed hepatoid adenocarcinoma of the stomach.

© 2021 Lin JX et al. *JAMA Network Open*.

eTable 3. Univariable and Multivariable Analyses of Risk Factors Associated With Overall Survival in Derivation Cohort Using Cox Mixed-Effects Model

**Variables**	**Univariable analysis**			**Multivariable analysis**	
	**HR (95%CI)**	**P value**		**HR (95%CI)**	**P value**
Age, yr					
<60	Ref				
≥60	1.042 (0.662 - 1.640)	.86			
Sex					
Male	Ref				
Female	0.734 (0.424 - 1.270)	.27			
BMI					
<25	Ref				
≥25	0.723 (0.421 - 1.241)	.24			
ASA scores		.88			
I	Ref				
II	0.987 (0.568 - 1.714)	.96			
III	1.095 (0.482 - 2.484)	.83			
Tumor locations		.91			
Upper	Ref				
Middle	0.792 (0.398 - 1.577)	.51			
Lower	0.831 (0.482 - 1.433)	.51			
Mix	1.201 (0.616 - 2.342)	.59			
Tumor size, cm					
<5	Ref				
≥5	1.212 (0.776 - 1.894)	.40			
Lymphovascular invasion					
Absent	Ref			Ref	
Present	2.790 (1.639 - 4.751)	**<.001**		1.332 (0.704 - 2.521)	.36
Perineural invasion					
Absent	Ref			Ref	
Present	2.883 (1.807 - 4.600)	**<.001**		2.128 (1.274 - 3.554)	**.009**
AFP, ng/mL[#]					
<20	Ref				
≥20	1.127 (0.653 - 1.944)	.67			
CEA, ng/mL					
<5	Ref			Ref	
≥5	2.333 (1.496 - 3.639)	**<.001**		1.718 (1.076 - 2.744)	**.03**
CA19-9, U/mL[#]					
<37	Ref				
≥37	1.290 (0.629 - 2.643)	.49			
pT category		**.003**			.39
T1	Ref			Ref	

© 2021 Lin JX et al. *JAMA Network Open*.

T2	1.118 (0.265 - 4.721)	.88		0.909 (0.210 - 3.925)	.90
T3	2.542 (0.742 - 8.712)	.14		1.363 (0.376 - 4.944)	.64
T4a	3.567 (1.086 - 11.719)	.04		1.452 (0.408 - 5.162)	.56
T4b	3.041 (0.787 - 11.751)	.11		1.635 (0.401 - 6.666)	.49
pN category		**<.001**			**.01**
N0	Ref			Ref	
N1	1.699 (0.655 – 4.407)	.28		1.152 (0.427 - 3.104)	.78
N2	2.622 (1.117 - 6.153)	.03		1.603 (0.652 - 3.938)	.30
N3a	3.458 (1.457 - 8.207)	.005		1.444 (0.542 - 3.849)	.46
N3b	7.782 (3.205 - 18.898)	<.001		3.715 (1.337 - 10.323)	.01
Examined lymph nodes					
<16	Ref				
≥16	1.406 (0.711 - 2.783)	.33			
Distant metastasis					
Absent	Ref				
Present	1.527 (0.863 - 2.704)	.15			
Type of resection					
Curative surgery	Ref				
Palliative surgery	1.715 (0.818 - 3.594)	.15			
Neoadjuvant chemotherapy					
No	Ref				
Yes	1.962 (0.894 - 4.306)	.09			
Adjuvant chemotherapy					
No	Ref				
Yes	0.874 (0.553 - 1.381)	.57			
Histological type					
SHAS	Ref				
MHAS	1.166 (0.742 - 1.831)	.51			

#Ninety-two AFP and 65 CA19-9 were not recorded.

Abbreviations: HR, hazard ratio; CI, confidence interval; Ref, reference; BMI, body mass index; ASA: American Society of Anesthesiologists; AFP, alpha-fetoprotein; CEA, carcinoembryonic antigen; CA19-9, carbohydrate antigen 19-9; pT, pathological tumor; pN, pathological node; SHAS, simple hepatoid adenocarcinoma of the stomach; MHAS, mixed hepatoid adenocarcinoma of the stomach.

© 2021 Lin JX et al. *JAMA Network Open*.

eTable 4. Comparison of Concordance Index and Akaike Information Criterion of Nomogram, AJCC pTNM Staging System, and Clinical Model Between Valication Cohort (FMUUH) and Derivation Cohort (Other 13 Centers)

	C-index (95% CI)	C-index changed (95% CI)	P	AIC
**Derivation cohort**				
Nomogram	0.709(0.651-0.767)	-	-	818.060
pTNM stage	0.627(0.572-0.683)	0.081(0.036-0.122)	**<.001**	841.721
Clinical model#	0.623(0.566-0.679)	0.086(0.045-0.140)	**<.001**	843.718
**Validation cohort**				
Nomogram	0.738(0.621-0.854)	-	-	149.992
pTNM stage	0.690(0.573-0.806)	0.048(-0.050-0.106)	.23	156.803
Clinical model#	0.694(0.576-0.811)	0.044(-0.056-0.122)	.34	158.796

#Clinical model：pTNM stage + adjuvant chemotherapy.

Abbreviations: C-index, concordance index; AIC, Akaike's information criterion; CI, confidence interval; pTNM, pathological tumor-node-metastasis; FMUUH: Fujian Medical University Union Hospital.

© 2021 Lin JX et al. *JAMA Network Open*.

eTable 5. Comparison of Concordance Index and Akaike Information Criterion of Nomogram, AJCC pTNM Staging System, and Clinical Model Between Simple and Mixed HAS Groups

	**C-index (95% CI)**	**C-index changed (95% CI)**	**P**	**AIC**
**SHAS type**				
Nomogram	0.712(0.638-0.785)	-	**-**	415.553
pTNM stage	0.623(0.547-0.700)	0.088(0.017-0.145)	**.007**	440.273
Clinical model#	0.625(0.546-0.704)	0.087(0.018-0.164)	**.02**	442.171
**MHAS type**				
Nomogram	0.715(0.649-0.781)	-	**-**	511.899
pTNM stage	0.650(0.585-0.715)	0.065(0.001-0.111)	**.02**	522.363
Clinical model#	0.653(0.589-0.718)	0.062(0.001-0.111)	**.03**	524.349

#Clinical model：pTNM stage + adjuvant chemotherapy.

Abbreviations: C-index, concordance index; AIC, Akaike’s information criterion; CI, confidence interval; pTNM, pathological tumor-node-metastasis; SHAS, simple hepatoid adenocarcinoma of the stomach; MHAS, mixed hepatoid adenocarcinoma of the stomach.

© 2021 Lin JX et al. *JAMA Network Open*.

eTable 6. Demographic and Clinicopathological Characteristics of Low-Risk and High-Risk Groups

**Characteristics**	**Low-risk group (n=185)**	**High-risk group (n=125)**	**P**
**Clinical Factors**			
Age, yr, mean±SD	62.8±9.4	60.7±11.2	.09
BMI, kg/m^2, mean±SD	22.8±3.1	22.5±2.9	.29
Sex			.09
Male	147(79.5)	89(71.2)	
Female	38(20.5)	36(28.8)	
ASA scores			.59
I	35(18.9)	25(20.0)	
II	126(68.1)	83(66.4)	
III	21(11.4)	12(9.6)	
Unknown	3 (1.6)	5 (4.0)	
Tumor locations			**.04**
Upper	57(30.8)	51(40.8)	
Middle	21(13.0)	22(17.6)	
Lower	80(43.2)	34(27.2)	
Mix	24(13.0)	18(14.4)	
Tumor size, cm, mean±SD	4.9±2.5	6.2±2.6	**<.001**
AFP, ng/mL, median (IQR) #	73.3 (4.9-479.6)	68.8 (3.3-1000.0)	.83
CEA, ng/mL, median (IQR)	2.5 (1.5-4.4)	5.3 (2.0-15.3)	**<.001**
CA19-9, U/mL, median (IQR) #	8.6 (4.7-17.9)	10.7 (6.2-20.0)	.09
**Treatment Factors**			
Type of resection			**.02**
Total gastrectomy	83(44.9)	72(57.6)	
Distal gastrectomy	77(41.6)	34(27.2)	
Proximal gastrectomy	19(10.3)	10(8.0)	
Palliative surgery	6(3.2)	9(7.2)	
Neoadjuvant chemotherapy			.26
Yes	15(8.1)	6(4.8)	
No	170(91.9)	119(95.2)	
Adjuvant chemotherapy			.98
Yes	90(48.6)	61(48.8)	
No	95(51.4)	64(51.2)	
**Pathological Factors**			
Examined lymph nodes, mean±SD	27.4±12.6	35.5±15.5	**<.001**
Metastatic lymph nodes,	2.5±2.8	12.7±9.5	**<.001**
Lymphovascular invasion			**<.001**
Absent	115(62.2)	16(12.8)	
Present	70(37.8)	109(87.2)	
Perineural invasion			**<.001**

© 2021 Lin JX et al. *JAMA Network Open*.

Absent	162(87.6)	21(16.8)	
Present	23(12.4)	104(83.2)	
pT category			**<.001**
T1	21(11.4)	3(2.4)	
T2	34(18.4)	4(3.2)	
T3	49(26.5)	36(28.8)	
T4a	68(36.8)	72(57.6)	
T4b	13(7.0)	10(8.0)	
pN category			**<.001**
N0	56(30.3)	1(0.8)	
N1	55(29.7)	6(4.8)	
N2	52(28.1)	31(24.8)	
N3a	16(8.6)	54(43.2)	
N3b	6(3.2)	33(26.4)	
pTNM stage			**<.001**
IA	13 (7.0)	0 (0)	
IB	21 (11.4)	0 (0)	
IIA	21 (11.4)	0 (0)	
IIB	31 (16.8)	7 (5.6)	
IIIA	57 (30.8)	27 (21.6)	
IIIB	16 (8.6)	42 (33.6)	
IIIC	6 (3.2)	27 (21.6)	
IV	20 (10.8)	22 (17.6)	
Liver metastasis			.34
Yes	16(8.6)	15 (12.0)	
No	169(91.4)	110 (88.0)	
Histological type			.40
SHAS	83 (44.9)	50 (40.0)	
MHAS	102 (55.1)	75 (60.0)	

[#]Ninety-two AFP and 65 CA19-9 were not recorded.

Abbreviations: SD, standard deviation; BMI, body mass index; ASA: American Society of Anesthesiologists; AFP, alpha-fetoprotein; CEA, carcinoembryonic antigen; CA19-9, carbohydrate antigen 19-9; IQR, interquartile range; pT, pathological tumor; pN, pathological node; pM, pathological metastasis; pTNM, pathological tumor-node-metastasis; SHAS, simple hepatoid adenocarcinoma of the stomach; MHAS, mixed hepatoid adenocarcinoma of the stomach.

© 2021 Lin JX et al. *JAMA Network Open*.

eFigure 1. Study Flow Diagram

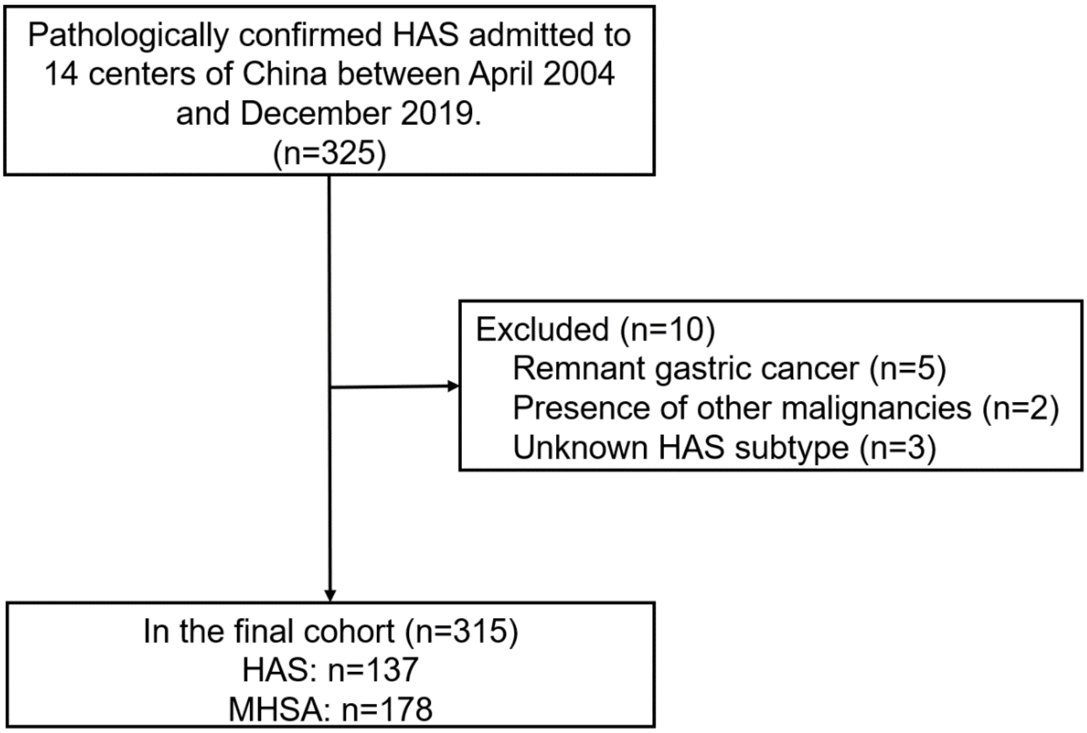

Abbreviations: HAS, hepatoid adenocarcinoma of the stomach; SHAS, simple hepatoid adenocarcinoma of the stomach; MHAS, mixed hepatoid adenocarcinoma of the stomach.

© 2021 Lin JX et al. *JAMA Network Open*.

eFigure 2. Histopathological Components of HAS

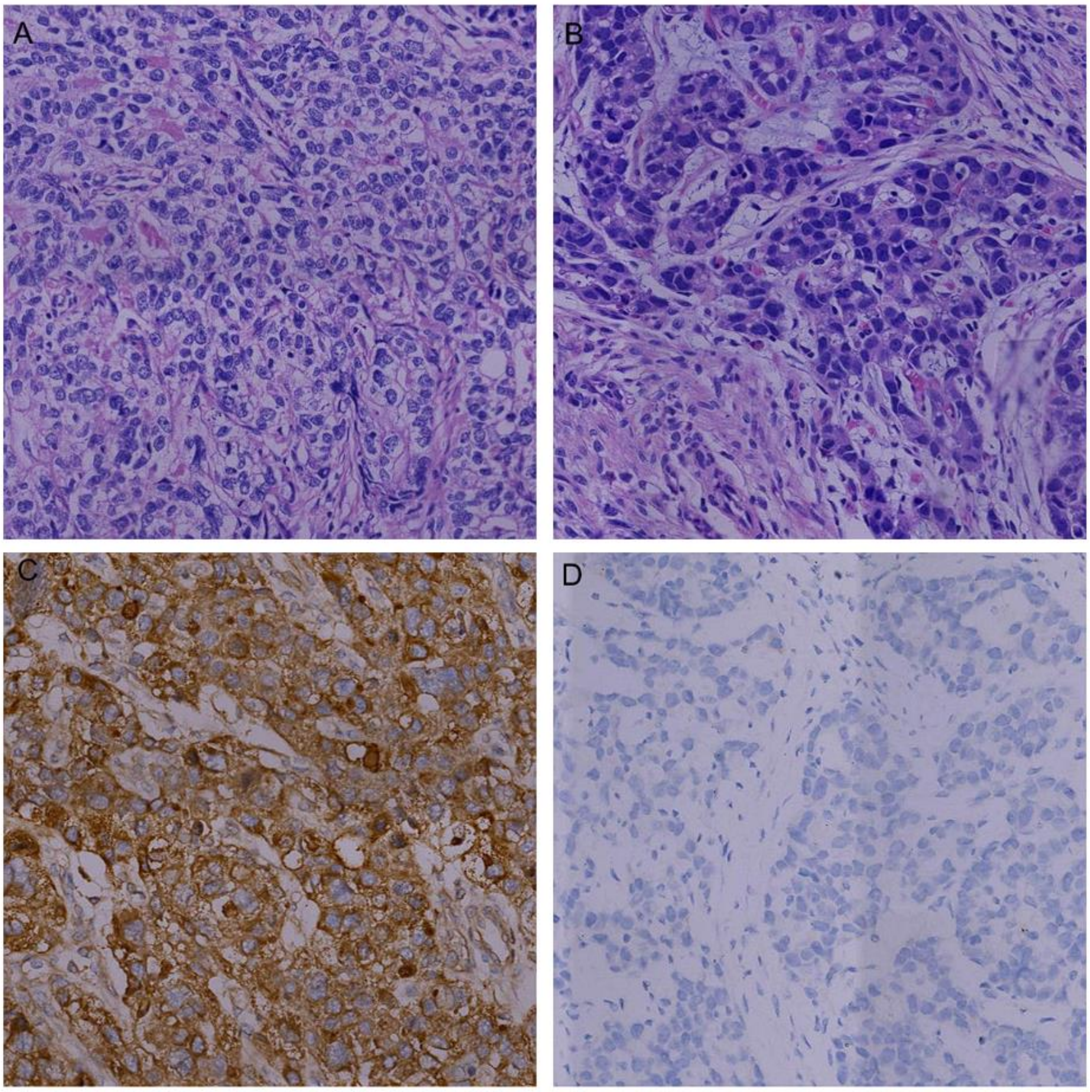

(A) Hepatoid component. (B) Common adenocarcinoma component. (C) AFP immunohistochemical staining positive. (D) AFP immunohistochemical staining negative. Abbreviations: HAS, hepatoid adenocarcinoma of the stomach; AFP, alpha-fetoprotein.

© 2021 Lin JX et al. *JAMA Network Open*.

eFigure 3. Calibration Curves for Overall Survival Nomogram Models in Derivation, Validation, and Whole Cohorts

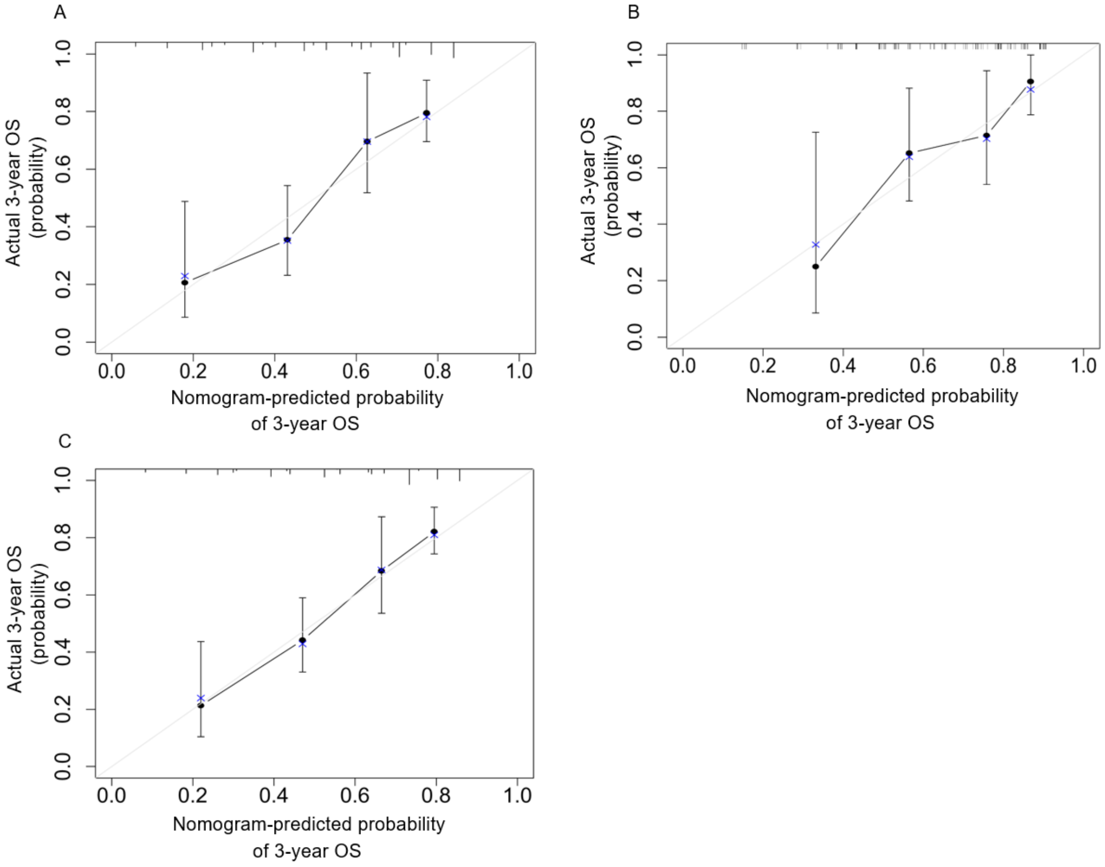

(A) Derivation cohort. (B) Validation cohort. (C) Whole cohort. The gray line represents ideal nomogram, and black line represents observed nomogram. A closer alignment with the grey line represents a better estimation. Vertical bars indicate 95% confidence interval, and crosses indicate bias-corrected estimates. Abbreviations: OS, overall survival.

© 2021 Lin JX et al. *JAMA Network Open*.

eFigure 4. Time-Dependent ROC Curve Comparing AUCs of Nomogram, AJCC pTNM Staging System, and Clinical Model in Derivation, Validation, and Whole Cohorts for Overall Survival of HAS

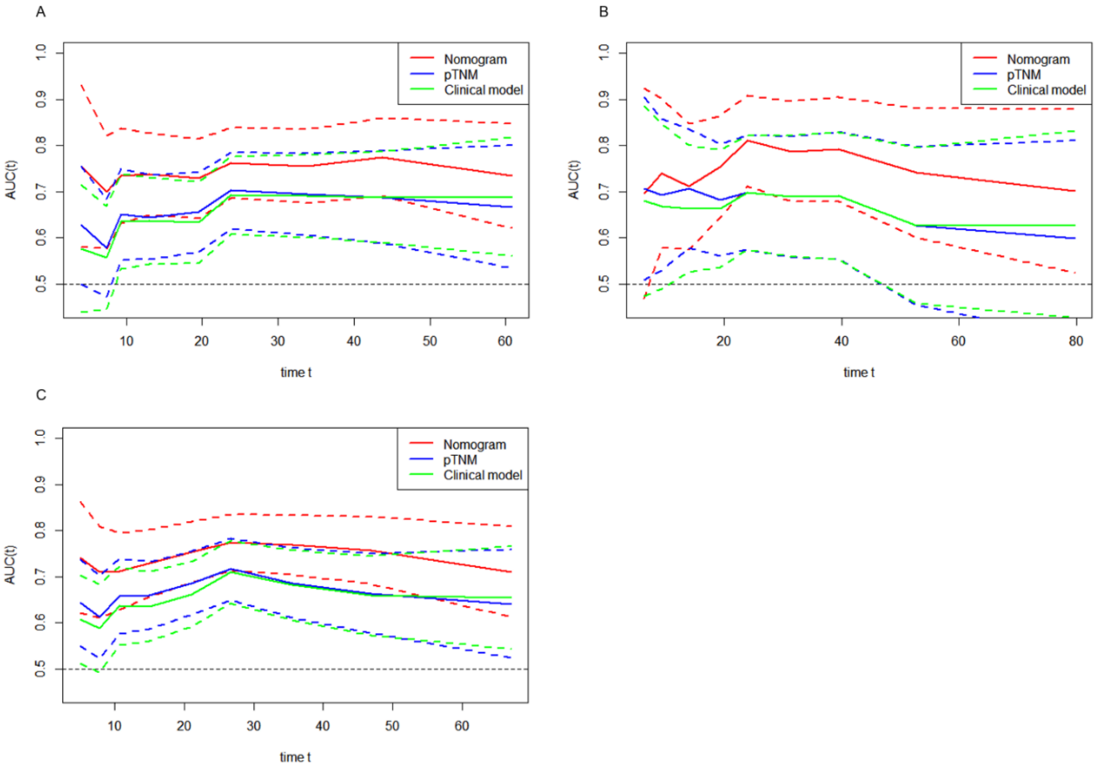

(A) Derivation cohort. (B) Validation cohort. (C) Whole cohort. Abbreviations: ROC, receiver operating characteristic; AUCs, area under the curves; pTNM, pathological tumor-node-metastasis; HAS, hepatoid adenocarcinoma of the stomach.

© 2021 Lin JX et al. *JAMA Network Open*.

eFigure 5. Decision Curve Analysis of Each Model for Estimating 3-Year Overall Survival in Derivation, Validation, and Whole Cohorts

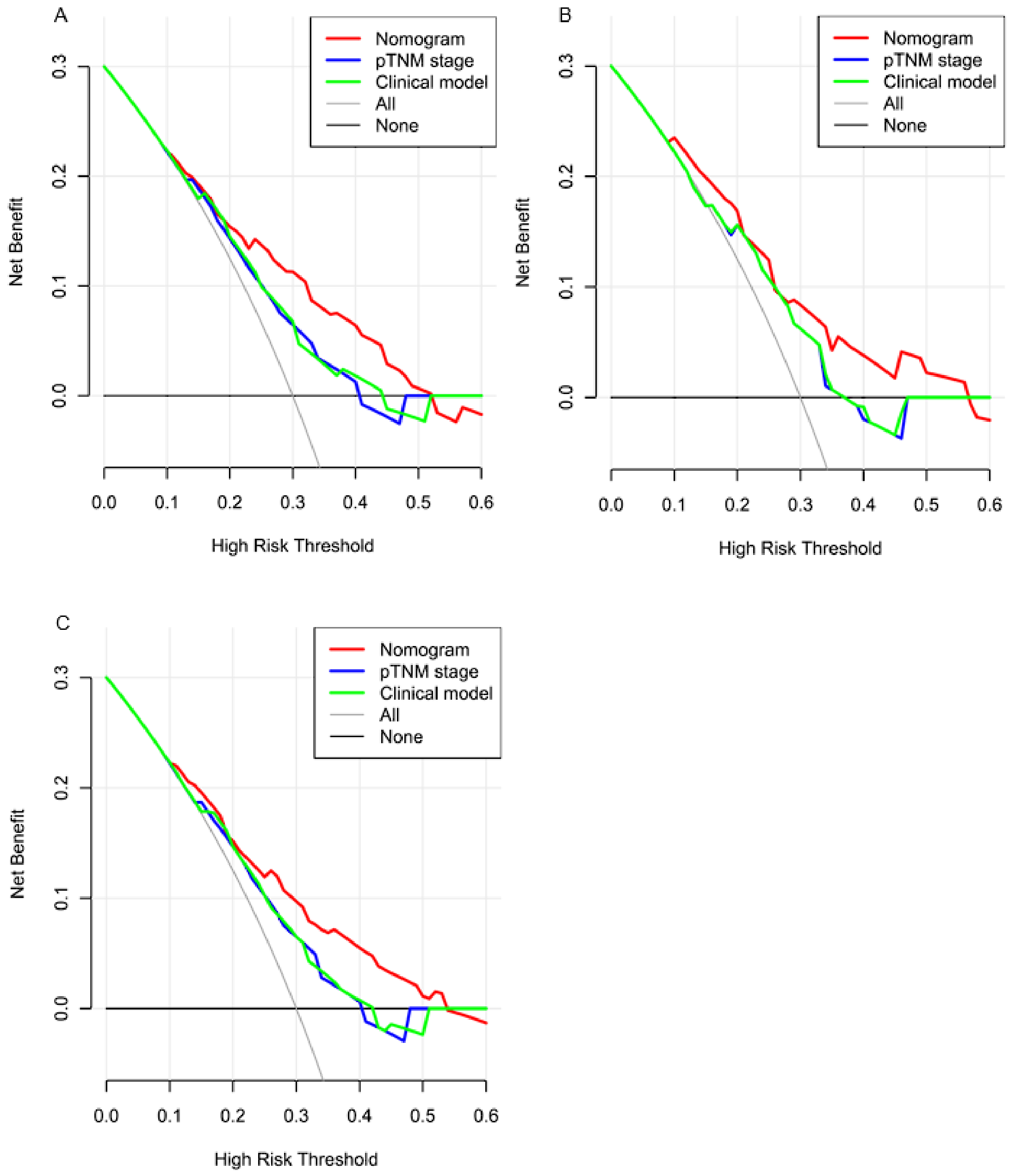

(A) Derivation cohort. (B) Validation cohort. (C) Whole cohort. Abbreviations: OS, overall survival. Abbreviation: pTNM, pathological tumor-node-metastasis.

© 2021 Lin JX et al. *JAMA Network Open*.

eFigure 6. Calibration Curves for Overall Survival Nomogram Models in Simple and Mixed HAS Groups

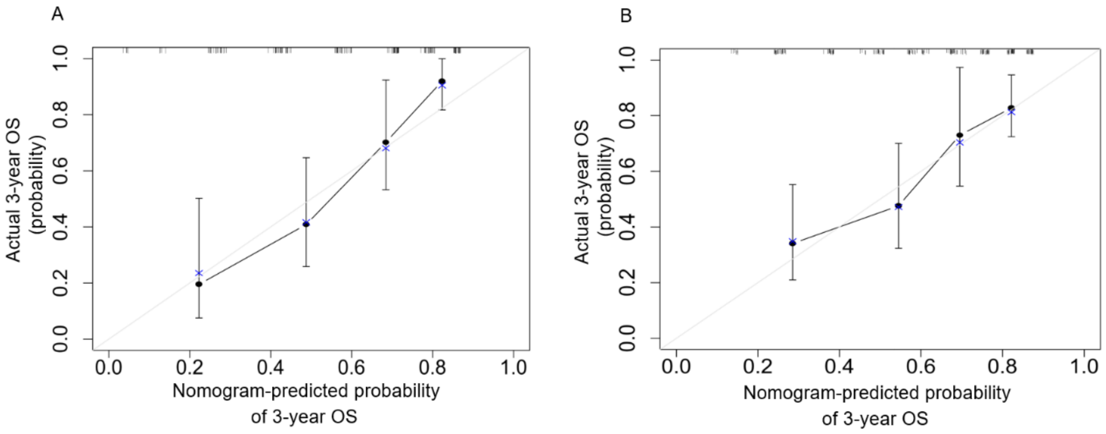

(A) SHAS group. (B) MHAS group. The gray line represents ideal nomogram, and black line represents observed nomogram. A closer alignment with the grey line represents a better estimation. Vertical bars indicate 95% confidence interval, and crosses indicate bias-corrected estimates. Abbreviations: SHAS, simple hepatoid adenocarcinoma of the stomach; MHAS, mixed hepatoid adenocarcinoma of the stomach.

© 2021 Lin JX et al. *JAMA Network Open*.

eFigure 7. Time-Dependent ROC Curve Comparing AUCs of Nomogram, AJCC pTNM Staging System, and Clinical Model in Simple and Mixed HAS Groups for Overall Survival

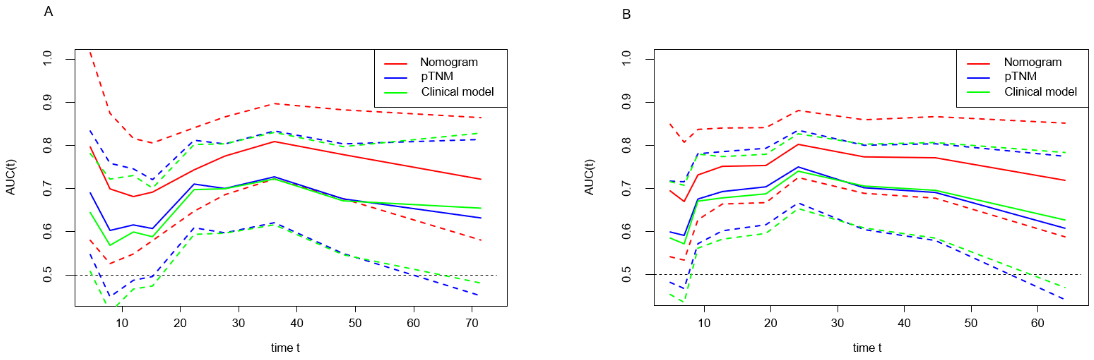

(A) SHAS group. (B) MHAS group. Abbreviations: ROC, receiver operating characteristic; AUC, area under the curve; pTNM, pathological tumor-node-metastasis; SHAS, simple hepatoid adenocarcinoma of the stomach; MHAS, mixed hepatoid adenocarcinoma of the stomach.

© 2021 Lin JX et al. *JAMA Network Open*.

eFigure 8. Decision Curve Analysis of Each Model for Estimating 3-Year Overall Survival in Simple and Mixed HAS Groups

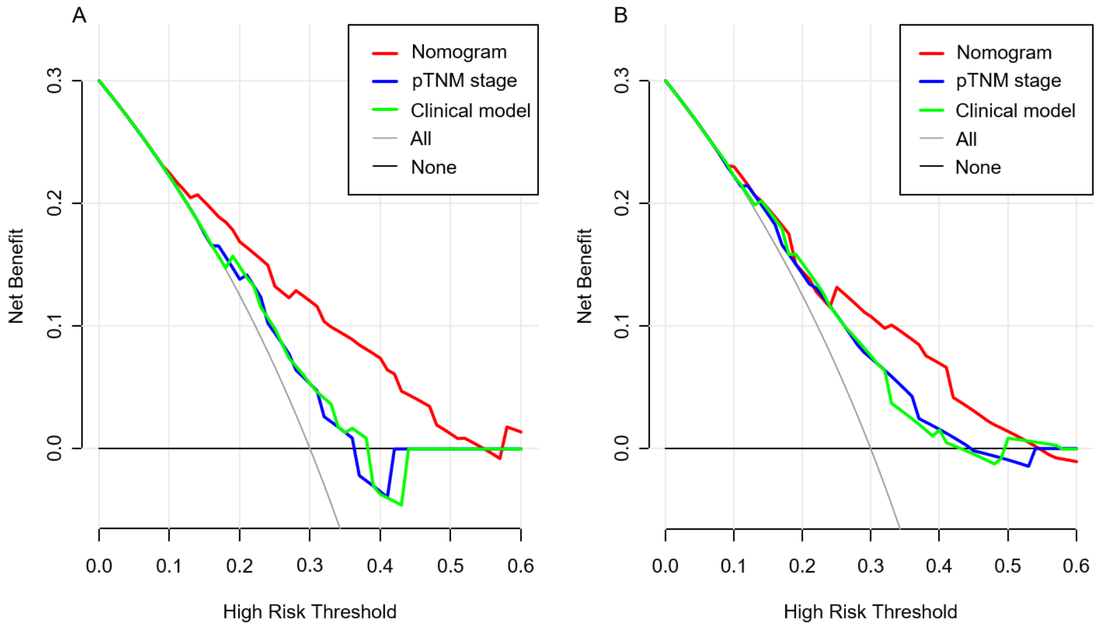

(A) SHAS group. (B) MHAS group. Abbreviations: pTNM, pathological tumor-node-metastasis; SHAS, simple hepatoid adenocarcinoma of the stomach; MHAS, mixed hepatoid adenocarcinoma of the stomach.

© 2021 Lin JX et al. *JAMA Network Open*.

eFigure 9. Division of Patients by Cutoff Points Produced by X-Tile Plot

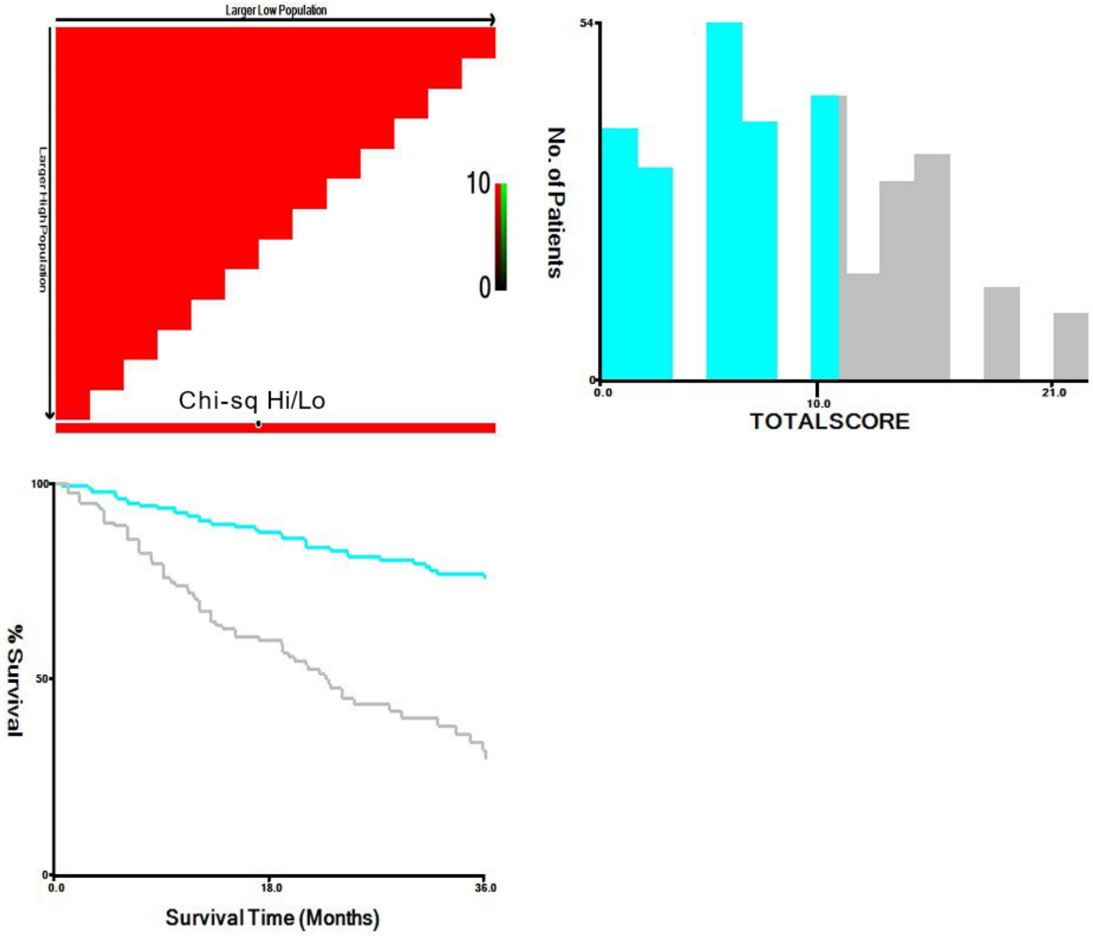

The produced log-rank chi-square value stratifies the patients into two groups by a cut-off total nomogram score of 10, showing a strong discriminatory capacity, with a chi-square value of 49.23 and a relative risk ratio of 1:2.87.

© 2021 Lin JX et al. *JAMA Network Open*.

eFigure 10. Kaplan-Meier Analysis of Overall Survival Between Low-Risk and High-Risk Groups by Histological Type

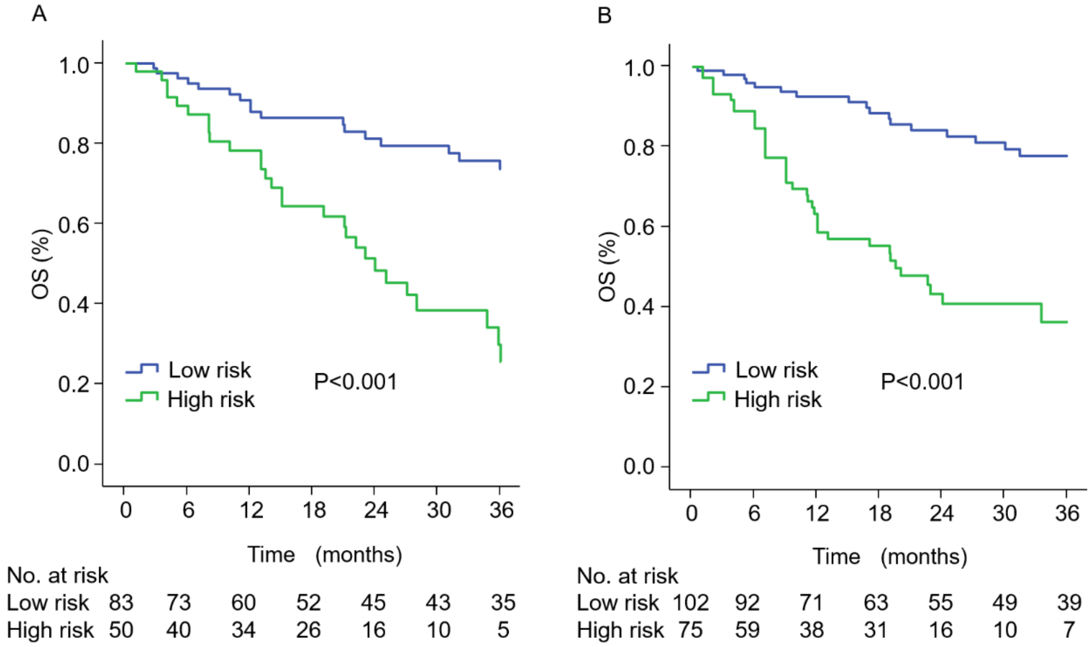

(A) SHAS group. (B) MHAS group. Abbreviations: ELNs, examined lymph nodes; OS, overall survival; HAS, hepatoid adenocarcinoma of the stomach; SHAS, simple hepatoid adenocarcinoma of the stomach; MHAS, mixed hepatoid adenocarcinoma of the stomach.

© 2021 Lin JX et al. *JAMA Network Open*.
